# Generation and Validation of miR-100 Hepatocyte-Specific Knock-Out Mice

**DOI:** 10.3389/fonc.2019.00535

**Published:** 2019-06-26

**Authors:** Dong Yang, Sai Tang, Yan Yang, Fan Yang, Wengang Jiang, Yakun Liu, Fengyun Zhang, Haoshu Fang, Siying Wang, Yuxia Zhang

**Affiliations:** Department of Pathophysiology, School of Basic Medical Science, Anhui Medical University, Hefei, China

**Keywords:** microRNA-100, Cre/LoxP, genotype, HCC, SHP-2

## Abstract

**Background:** Inactivation of microRNA-100 (miR-100) is involved in hepatocellular carcinoma (HCC) and miR-100 behaves as a tumor suppressor. To understand miR-100 function in HCC genesis and development *in vivo*, we developed hepatocyte-specific miR-100 deficient mice.

**Methods:** Mice homozygous for floxed miR-100 allele that carried the Alb-Cre transgene (miR-100^flox/flox^Alb -Cre^+^) were developed by mating miR-100^flox/flox^ mice with Alb-Cre^+/+^mice. The mice tails DNA were genotyped using the primers for LoxP sites and Cre recombinase, respectively. The specific deletion of miR-100 in the livers was verified by quantitative Real-time PCR (qRT-PCR). HE-staining was performed for histology analysis. Liver function was assessed by transaminase activity. The metabolic profiles of the hepatocytes were detected using a Seahorse XFe24 extracellular flux analyzer. The direct targets of miR-100 (such as IGF1R-β, mTOR and CDC25A) and HCC related protein (SHP-2) were detected by qRT-PCR and Western blot in liver tissues.

**Results:** The resultant homozygous knockout mice with genotype of miR-100^flox/flox^-Alb-Cre^+^ showed an 80% decrease in hepatic miR-100 expression. In adult mice, miR-100 knockout has no effect on the liver function and morphology. In aged mice, HE staining showed that miR-100 knockout caused infiltration of inflammatory cells and expansion of hepatocellular nuclei. Consistently, liver function was impaired in miR-100 knockout aged mice as indicated by increased serum AST and ALT levels. The metabolic analysis demonstrated that the miR-100 knockout hepatocytes tend to adopt glycolysis. The expressions of the miR-100 target genes, such as IGF1R-β, CDC25A and mTOR, were increased. In addition, the known HCC related protein, SHP-2 also was up-regulated in the knockout livers.

**Conclusions:** We successfully generated a miR-100 hepatocyte-specific knock-out mouse model. The malignant transformation related to HCC were observed in aged mice. Therefore, this model is suitable for investigating the mechanism of miR-100 inactivation contributing to HCC genesis *in vivo*.

## Introduction

MicroRNAs (miRNAs, miR) are small (19–24 nt) non-coding RNAs, that participate in key post-transcriptional regulation of target genes and most commonly lead to translational repression and/or degradation of the target mRNAs. More than 1,000 different miRNAs express in various tissues and each miRNA targets hundreds of protein-coding genes (1, 2). MiRNAs have been shown to be involved in various biological processes, such as cell differentiation, proliferation, apoptosis, motility (3, 4). Aberrant expression of miRNAs has been shown to act as tumor promoter or suppressor, depending on tumor types, and their targets in different cells (5–7).

MiR-100 is a member of the miR-99 family locating at 11q24.1 (Gene ID: 406892) (8, 9). Interestingly, its dual effects in diverse human cancers have been proposed in recent studies (10–12). Chen et al. have revealed that reduced expression of miR-100 in HCC tissues compared with matched paracancerous tissues. In addition, the inactivation of miR-100 was found to be correlated with higher incidence of lymph node metastasis, advanced TNM stage, and poor prognosis of HCC patients (13). Zhou et al. reported that miR-100 suppress HCC metastasis by down-regulation of Angiopoietin-2 (Angpt2) (14). Beyond HCC, downregulation of miR-100 occurs in breast cancer (15, 16), pancreatic adenocarcinoma (17), and ovarian cancer (18). Conversely, upregulation of miR-100 has been observed in HCC (19), small cell lung cancer (SCLC) (20), non-small cell lung carcinoma (NSCLC) (21), and renal cell carcinoma (RCC) (22), where it acts as a powerful tumor promoter.

We previously showed that inactivation of miR-100 promoting arsenic-induced EMT of BEAS-2B cells, suggesting a tumor-suppressing role in the initiation of lung cancer (23). Consistently, our recent work demonstrated that miR-100 expressed in normal hepatocyte cell line, L02, and specific inhibition (micro-RNA sponge) of miR-100-3p significantly enhanced malignant biological behavior and upregulated the expression of IGF1R-β and SHP-2 in this cell line (unpublished data). Overexpression of SHP-2 (24) and IGF1R-β (25) are associated with multiple human tumors including HCC. To dissect in more detail, the potential role of miR-100 in genesis, progress of HCC *in vivo*, one strategy would be to develop a mouse model with liver-specific deletion of miR-100. To achieve this, the Cre/LoxP system targeting strategy was used. We generated and validated hepatocyte-specific miR-100-knockout mice by crossing miR-100^flox/flox^ mice with Alb-Cre mice (Cre recombinase under the control of the adenovirus Alb promoter, allowing expression of Cre exclusively in hepatocytes). Our initial studies using the mice model have shown that miR-100 knockout results in early malignant transformation of HCC. Some known target genes of miR-100 such as IGF1R-β and HCC related protein, SHP-2 are up-regulated.

## Methods

### Mouse Strains

**Figure 1:**
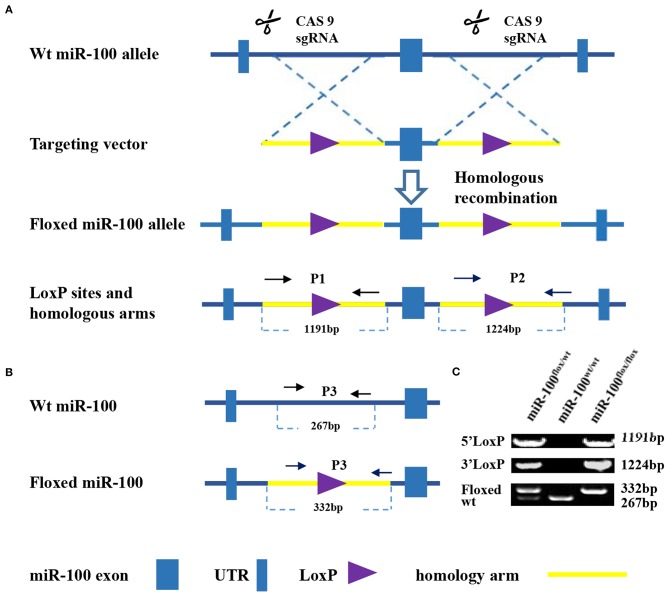
Strategy for generation of a condition knockout of miR-100. **(A)** Schematic diagram of the wild type mir-100 allele (top), the miR-100 gene targeting vector (middle), the floxed miR-100 allele (bottom). Two loxP sites (purple triangle) flanking miR-100 exon (blue square) within 5′ and 3′ homology arms (yellow, 1191 and 1,124 kb, respectively) are indicated. **(B)** Multiple PCR based genotyping. Primers with the expected product size are indicated. Primers 1 and 2 were used to detect the presence of 5′ (1,191 bp) and 3′ (1,224 bp) homologous arms of the targeted miR-100 allele. Primers 3 were used to distinguish floxed miR-100 allele (332 bp) from the wild-type allele (267 bp). **(C)** Amplified fragments having sizes specific for the wild-type and floxed miR-100 alleles were obtained of wild-type (miR-100^wt/wt^), heterozygous (miR-100^flox/wt^), and homozygous (miR-100^flox/flox^) mice.

C57BL/6 homozygous floxed miR-100 mice (bearing loxP sites flanking the only exon of the miR-100 gene, Figure 1A) was constructed at Model Animal Research Center of Nanjing University (12 Xuefu Road, Pukou District, Nanjing, 210008). And we have independent copyright for this mouse strain. C57BL/6 Alb-Cre mice (Cre expression is controlled by the promoter of the hepatocyte-specific gene Albumin), described previously (26), were purchased from Model Animal Research center of Nanjing University. All mice were housed in a SPF environment at Experimental Animal Center of Anhui Medical University. Collecting organs and tissues from mice following euthanasia using CO_2_ were approved by Anhui Medical University Institutional Animal Care and Use Committee (IACUC) and Ethics Committee.

### Generation of miR-100 Conditional Knockout Mice

**Figure 2:**
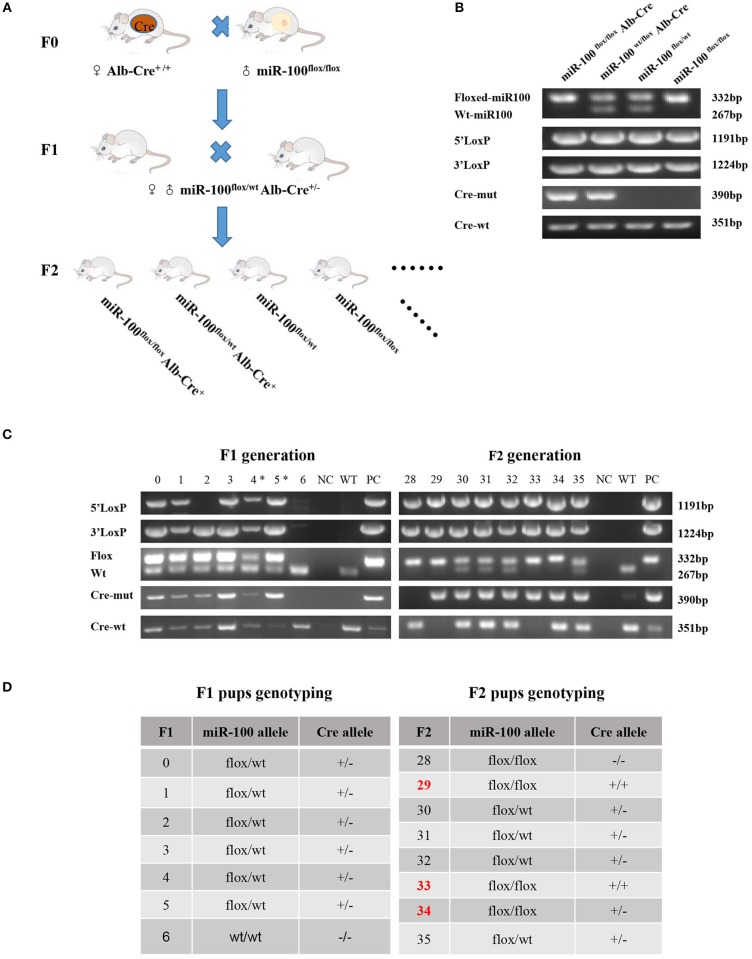
Generation of mice with Alb-Cre mediated miR-100 deletion. **(A)** Strategy for mice mating to generate mice bearing homozygous floxed miR-100 and cre-mut simultaneously. **(B)** PCR-based genotyping showing DNA products having sizes specific for the floxed allele, Cre allele as well as 5′ and 3′ homology arms in tail DNA. **(C)** Representative genotyping of F1 (left) and F2 (right) generation. **(D)** The corresponding genotypes of F1 and F2 mice are shown, miR-100^flox/flox^ Alb-Cre^+^ mice were selected as miR-100 knockout mice. F2 mice resulted from breeding pair of #4 F1 female and #5 male, designated with (*).

The mating scheme was similar to one described by Tan et al. (27). In brief, homozygous floxed miR-100 mice (miR-100^flox/flox^) were crossed with Alb-Cre^+/+^ mice and the progeny carrying a floxed miR-100 allele and Alb-Cre allele were inbred (Figure 2A). This led to miR-100^flox/flox^Alb-Cre^+^ mice (hereafter designated as homozygous). The following genotypes: miR-100^flox/wt^Alb-Cre^+^ (hereafter designated as heterozygous) and miR-100^flox/flox^ are used as controls in all experiments.

### Genotyping

Offspring genotype was identified by PCR analysis of tail DNA for the LoxP sites flanking the miR-100 gene and the Cre transgene (also referred to as Cre-mutant in this paper). Sequences of forward and reverse primers for miR-100 alleles and the Alb-Cre transgene are list in Table 1.

**Table 1:** Primers sequences of PCR used in this study.

**NO**	**Primer name**	**Primer sequence**	**Band size**	**Purpose**
P1	miR-100-loxp F1	CTTCTTCCTGTGCCATCAGCTCAG	Flox = 1,191 bp	To detect 5′-LoxP site
	miR-100-loxp R1	AAGTTATGTGCACGAATTCTGGTTC	Wt = none	
P2	miR-100-loxp F2	ATGGACTAACAGAAGAACCCGTTGTG	Flox = 1,224 bp	To detect 3′-LoxP site
	miR-100-loxp R2	CGTGTAAATCAGGCCAAGTAGACTG	Wt = none	
P3	miR-100-wt-t F	GTGGTAAGGCTGTTCAAAGGCAG	Flox = 332 bp	Rapid identification of homozygotes
	miR-100-wt-t R	CTGAAGGGGATAAGGTTGCCTCTC	Wt = 267 bp	
P4	miR-100 del -F	TCTGGGCT AGCAAGTAAATGTC	miR-100 KO = 467 bp	To detect miR-100 KO allele
	miR-100 del -R	CTGTCAGCCAGTCTTCACTTTCTG	miR-100 wt = 1,453 bp miR-100 Flox = 1,585 bp	
P5	Alb-Cre-F1	TGCAAACATCACATGCACAC	P5 /P6 for Wt = 351 bp	To detect Cre
P6	Alb-Cre-R1	TTGGCCCCTTACCATAACTG	P7 /P6 for Mut = 390 bp	
P7	Alb-Cre-F2	GAAGCAGAAGCTTAGGAAGATGG		

Primers P1, P2, P3 are used to genotype miR-100 alleles (miR-100^wt^, miR-100^flox^). The P1 primers allow to detect the presence of 5′LoxP site and 5′ homogenous arm and the P2 primers allow to detect 3′ LoxP site and 3′ homogenous arm. The P3 primers allow to amplify either the wild type miR-100 allele or the presence of the LoxP site, which are used to identify homozygous for floxed miR-100 rapidly (Figure 2B). Sequences of P1, P2, and P3 primers are list in Table 1.

**Figure 3:**
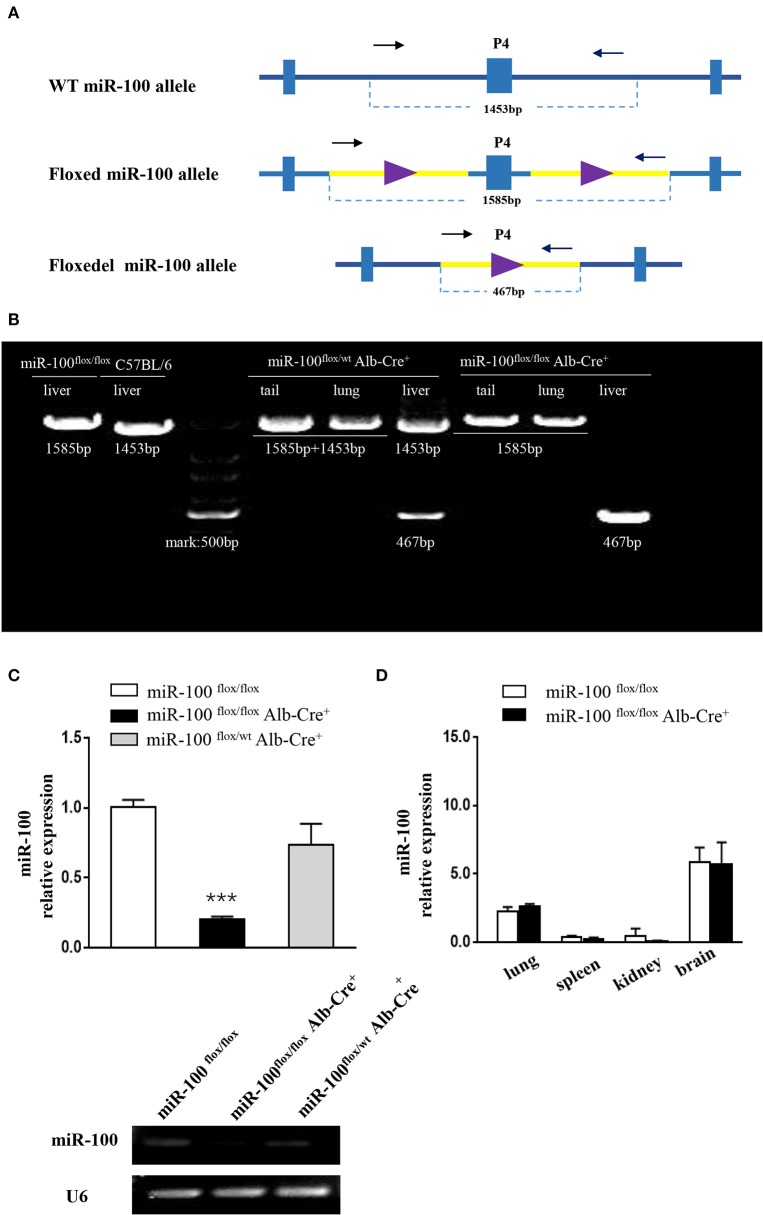
Validation of liver-specific knockout mir-100 in miR-100^flox/flox^Alb-Cre^+^ mice. **(A)** Strategy for genotyping of miR-100 floxdel allele in the livers. Primers designed for amplifying either the wild type (1,453 bp) or the floxed (1,585 bp) and knockout (i.e., miR-100 floxdel, 467 bp) miR-100 allele. **(B)** Examples of genotyping are shown for the miR-100 floxdel allele. Genomic DNA was collected from tails, lungs, and livers from indicated mice, and amplified using P4 primers. Fragments for miR-100 knockout (467 bp) were amplified exclusively in the liver not in other tissues. DNA fragment size of the wild type (1,453 bp) or the floxed (1,585 bp) miR-100 allele are indicated. **(C)** Quantitative RT-PCR confirmed miR-100 deletion in the liver of homozygous mice. Analysis of liver miR-100 expression in miR-100^flox/flox^ Alb-Cre^+^ and control mice were performed. The expression level was normalized to that of U6 (*n* = 3 per group). Data are representative of 3 independent experiments. ***P < (*n* = 3 per group). Data are representative of 3 independent experiments. ***P < 0.0001 (Student's *t*-test). **(D)** miR-100 expression in extra-hepatic organs in miR-100 ^flox/flox^ Alb-Cre^+^ and control mice.

P4 primers are used to determine whether the floxed area is deleted specifically (referred to as miR-100^floxdel^, 467 bp) in the liver (Figure 3A).

Alb-Cre transgenic mice genotyping were determined using the primers P5, P6 and P7. P5 /P6 are used for detecting Cre^wt^, which amplify a 351 bp fragment; P7/P6 are used for detecting Cre^mut^, which amplify a 390 bp fragment (Figure 2B). Sequences of P4, P5, P6, and P7 primers are list in Table 1.

The PCR procedure was performed under the following conditions: denaturation at 95°C for 5 min; 35 cycles of 95°C for 30 s; followed by annealing at 50°C (flox-PCR) or 62°C (Cre-PCR) for 30s and extension at 72°C for 30 s; and a completion step at 72°C for 3 min; and 4°C hold.

The PCR mixture containing 10 ul Taq MasterMix (Taq polymerase, dNTPs), 0.5 ul forward primer and reverse primer, respectively, 5μl ddH_2_O, 4μl genomic DNA was performed in total volume of 20 μl.

### Quantitative Real Time PCR (qRT-PCR)

The total RNA was extracted from mouse liver, lung, spleen, kidney, brain tissue (stored at −80°C) using TRIzol reagent (Invitrogen) following the manufacturer's instructions. RNA purity and concentration were determined using a Multiskan™ FC reader (Thermo Fisher Scientific, USA). For miR-100 analysis, the cDNA was synthesized with random primers using a reverse transcription kit (Gene Pharma, Shanghai, China). Levels of mature miR-100 were measured using a Hairpinit TM miRNA qPCR Quantitation Kit (Gene Pharma, Shanghai, China) according to manufacturer's protocol. The small nuclear RNA U6 served as an internal control. For mRNA analysis, the cDNA was synthesized using a PrimerScript™ RT reagent kit (TaKaRa, China) and random primers (Sangon Biotech, Shanghai, China). Real-time PCR was performed using TB Green™ Premix Ex Taq™ II (TaKaRa, China) according to the manufacturer's instructions. β-actin was used as an internal control. The relative expression of genes was analyzed utilizing the 2^−ΔΔ^ Ct method. The sequences of primers were listed in Table 2.

**Table 2:** Primers sequences of qRT-PCR used in this study.

**Gene**	**Forward**	**Reverse**
Mmu-miR-100	ATCATTAAACCCGTAGATCCGAA	TATGGTTGTTCACCTCTCGTTCAC
U6	CAGCACATATACTAAAATTGGAACG	ACGAATTTGCCTGTCATCC
IGF1-β	GTGGGGGCTCGTGTTTCTC	GATCACCGTGCAGTTTTCCA
CDC25A	ACAGCAGTCTACAGAGAATGGG	GATGAGGTGAAAGGTGTCTTGG
mTOR	ACCGGCACACATTTGAAGAAG	CTCGTTGAGGATCAGCAAGG
SHP-2(PTPN11)	AGAGGGAAGAGCAAATGTGTCA	CTGTGTTTCCTTGTCCGACCT
β-actin	AGAGGGAAATCGTGCGTGAC	CAATAGTGATGACCTGGCCGT

### HE Staining

Formalin-fixed liver tissues were embedded in paraffin using standard procedures. Sections were then cut at 4 μm and stained with hematoxylin and eosin for routine histological examination. Histopathological changes were determined by a skilled pathologist.

### Determination of ALT/AST Levels

Serum aspartate aminotransaminase (AST) and alanine aminotransaminase (ALT) were measured as indicators of liver function. The levels of the enzymes were determined using a commercial kit (Jian Cheng, Nanjing, China) according to the manufacturer's instructions.

### Hepatocytes Isolation and Seahorse Metabolic Flux Analysis

Under ether anesthesia, hepatocytes were isolated from miR-100^flox/flox^ and miR-100^flox/flox^Alb-Cre^+^ mice using a modified version of a previously described two-step collagenase perfusion procedure (28). The cell viability is higher than 90% determined by trypan blue exclusion test. Isolated hepatocytes were suspended in DMEM supplemented with 10% FBS, 100 units/ml of penicillin, 100 μg/ml of streptomycin and plated in cell culture dishes. Hepatocytes were allowed to establish monolayer in a humidified atmosphere containing 5% CO_2_ at 37°C for 2 h. Then the medium was replaced with a fresh DMEM and cells were incubated for up to 24 h. After incubation, the cells were used for the Seahorse metabolic flux analysis.

Cellular extracellular acidification rate (ECAR) was measured using a Seahorse XFe24 extracellular flux analyzer (Seahorse Bioscience). Experiments were performed according to the manufacturer's instructions. Protein standardization was performed after each experiment.

### Western Blot Analysis

Mouse tissues were crushed into a fine powder in liquid nitrogen and lysed in RIPA buffer supplemented with complete protease inhibitor (Roche, Switzerland). Protein concentration was determined using the BCA method (KeyGEN Bio TECH Nanjing, China). Western blot analysis was performed as previously described (23). Aliquots (20 μg) resolved by 8% SDS-PAGE gels electrophoresis, transferred to PVDF membrane (Pall, USA). After blocking with 5% non-fat milk in phosphate buffered saline/Tween-20, the membrane was incubated with antibodies specific to SHP-2 (1:1,000; #3397, Cell Signaling Technology, Boston, USA), CDC25A (1:1,000; #137353, Abcam, UK), mTOR (1:1,000; #2983, Cell Signaling Technology, Boston, USA), IGF1R-β (1:1,000; #9750, Cell Signaling Technology, Boston, USA). The antigen-antibody complexes were detected using an enhanced chemiluminescence (Advansta, Menlo Park, USA). The abundance assessed quantitatively using the Image J softwar.

### Statistical Analysis

Statistical analysis was performed using SPSS software (version 20). Reliability was calculated using Cronbach's alpha. All qPCR data are shown as mean ± SD from 3 mice each group. Data was analyzed using unpaired Student's *t*-test. A *P* value <0.05 was regarded statistically significant.

## Results

### Strategies for Generating Mice With a Floxed-miR-100 Allele

The strategy, illustrated in Figure 1A, was utilized to modify miR-100 genomic sequences by flanking miR-100 exon with two LoxP recombination sequences to generate a “floxed”-miR-100 allele.

PCR-based strategy for genotyping was shown in Figure 1B. Multiplex PCR using primers 1, 2, and 3 yield DNA products having sizes specific for the wild-type and floxed miR-100 alleles (Figure 1C). For homozygous (miR-100^flox/flox^) mice, an 1191-bp, a 1224-bp and a 332-bp fragment will be amplified. For heterozygous (miR-100^flox/wt^) mice, in addition to bands mentioned above, a 267-bp band will be detected. While a 267-bp fragment alone will be detected for genotype of wild-type (miR-100 ^wt/wt^).

### Generation of Mice With Alb-Cre Mediated miR-100 Deletion and Genotyping

Alb-Cre, a Cre line expressed in hepatocytes have the albumin promoter, which directs transcription of Cre-recombinase allowing deletion of floxed sequences in the liver (29). After miR-100^flox/flox^ mouse crossing with Alb-Cre homozygous mouse we get F1 generation, which haplotypes is miR-100^flox/wt^ Alb-Cre^+^. F1 were inbred, generating F2 with different genotypes, such as miR-100^flox/flox^Alb-Cre^+^, miR-100^flox/wt^Alb-Cre^+^, miR-100^flox/flox^ and miR-100^flox/wt^ (Figures 2A,B) and so on.

Examples of genotyping F1 and F2 mice were shown in Figure 2C and summarized in Figure 2D. Genotype of miR-100^flox/flox^ Alb-Cre^+^ (#33) or miR-100^flox/wt^Alb-Cre^+^ (#30, #31, #32, #35) mice showed simultaneously flox and cre in tail DNA. Liver-specific miR-100 knockout mice were screened based on Cre-positive as well as homozygous miR-100^flox/flox^ genotype, such as #29, #33, #34 (Figures 2C,D).

### Verification of Hepatocyte-Specific Knockout miR-100

To confirm miR-100 was deleted correctly in these mice, two approaches were employed. Firstly, P4 primers (referred to as “null” primers) were designed for amplify miR-100^floxdel^ allele (Figure 3A): fragments 467 bp for knockout-miR-100, 1,585 bp for floxed-miR-100 and 1,453 bp for wt-miR-100.We performed PCR analysis of DNA from a variety of tissues including tail, lung, and liver from #33 miR-100^flox/flox^Alb-Cre^+^ (homozygotes) mice (Figure 3B). As expected a 467 bp band that represents the deletion of miR-100 was exclusively detectable in the livers, but not in other tissues consistent with liver specificity of the Alb promoter. As a control, we also tested #35 heterozygotes (miR-100^flox/wt^Alb-Cre^+^), both 467 bp (miR-100 floxdel), and 1,453 bp (wt) fragments were detectable in the liver, while 1,453 bp (represent of wt-miR-100) and 1,585 bp (represent of floxed-miR-100) were amplified without the band of 467 bp (miR-100 floxdel) in the tail and the lung due to not having Cre recombinase in these tissues. A 1,585 and 1,453 bp band were alone amplified, respectively in the livers of miR-100^flox/flox^ (#28) and C57BL/6 mice. Notably, the 467 bp band was even stronger in the homozygous (#33) liver than that in the heterozygous (#35) liver. These data confirmed that Alb-Cre mediated miR-100 deletion exclusively occurred in the livers.

In addition, we examined the miR-100 expression in the liver tissue by qRT-PCR, which is the direct and credible way to detect whether miR-100 deletion was successful in the knockout mice. Livers from miR-100 ^flox/flox^ Alb-Cre^+^ (homozygous), miR-100 ^flox/wt^ Alb-Cre^+^ (heterozygous) as well as miR-100^flox/flox^ mice were analyzed. A reduction of miR-100 was observed in both knockout heterozygous and homozygous animals. Liver miR-100 was decreased by about 80% in homozygous (0.2 ± 0.02 vs. 1.0 ± 0.05, *P* < 0.0001) and by ~30% in heterozygous (0.73 ± 0.15 vs. 1.0 ± 0.05, *P* = 0.17) mice as compared with the littermate miR-100^flox/flox^ mice (Figure 3C, top). Agarose gel electrophoresis analysis of the same PCR reaction mixture clearly indicated the same results (Figure 3C, bottom). These results indicate that Cre-mediated recombination of the miR-100^flox^ allele indeed leads to a knockdown of miR-100. Some remnant miR-100 in homozygous mice is probably due to non-parenchymal liver cells continuing to express miR-100, which do not have Alb-promoter. This view was evidenced by undetectable miR-100 in the liver from miR-100^flox/flox^ EIIa-Cre^+^ mice, in which the adenovirus EIIa promoter directs expression of Cre recombinase in nearly in all tissues (Supplementary Figure 1). Additional qRT-PCR detection with other tissues such as lungs, spleens, kidneys, and brains were performed. There were no significant difference of miR-100 expression in these tissues of miR-100^flox/flox^ Alb-Cre^+^ mice compared with miR-100^flox/flox^ mice, confirming that miR-100 deletion was specific in the livers (Figure 3D).

Above data of both PCR amplification using “null” primers and qRT-PCR analysis of miR-100 in the liver tissue demonstrated that our genotyping strategy is credible and reliable.

### Phenotype in the Hepatocyte-Specific miR-100 Deletion Mice

**Figure 4:**
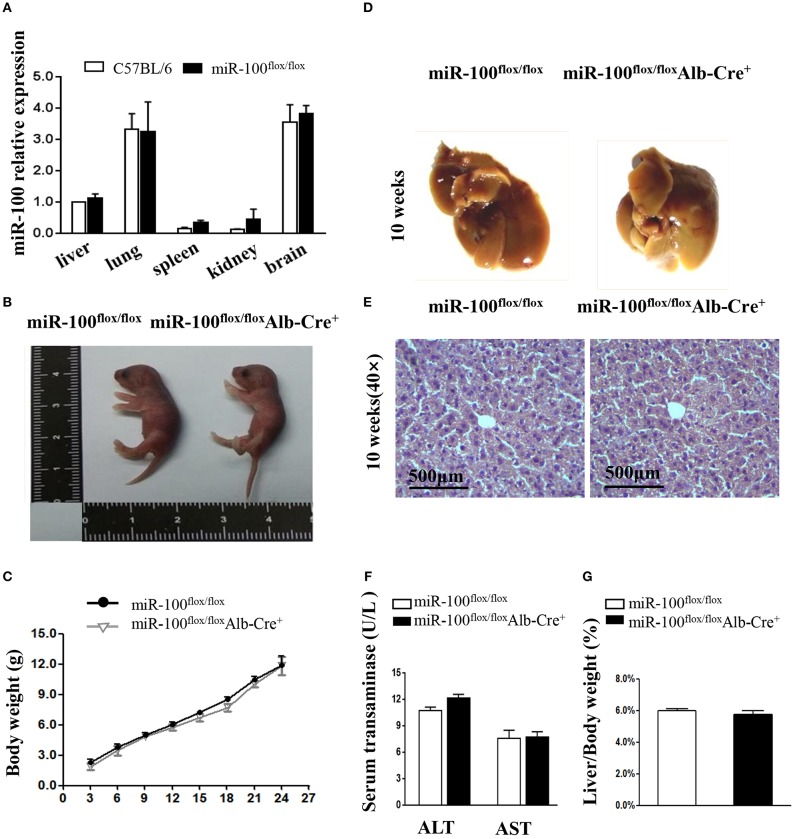
Phenotype analysis in the new born and adult hepatocyte-specific miR-100 deletion mice. **(A)** QRT-PCR analysis of miR-100 expression in the indicated tissues from miR-100^flox/flox^ and C57BL/6 mice. U6 was used as an internal control. The expression level was normalized and are presented relative to the level expressed in the liver. **(B)** Representative photographs of miR-100^flox/flox^Alb-Cre^+^ and miR-100^flox/flox^ newborn littermates. **(C)** Growth curve of miR-100^flox/flox^Alb-Cre^+^ and miR-100^flox/flox^ littermates. F2 pups were weighed every 3 days from postnatal day 3. Data are mean±SEM from 3 mice of each group. **(D–G)** Gross and histological analyses of livers from the 10-week-old miR-100 knock-out mice and control mice. Representative liver photographs **(D)** and liver tissues HE staining **(E)** were shown. Serum transaminase activity **(F)** and liver weitht/ body weight (LW/BW) ratios **(G)** of miR-100^flox/flox^ Alb-Cre^+^ and miR-100^flox/flox^ littermates were measured. Data are mean±SEM from each group (*n* = 3/group). Scale bars represent 500 μm.

The miR-100 expression in tissue samples of liver, lung, spleen, kidney, and brain was tested by qRT-PCR. Its expression varies greatly in different organs of C57BL/6, with higher expression in the lungs and brains, lower expression in the spleen and kidney and moderate expression in the livers. Homozygous miR-100^flox/flox^ mice are fertile and miR-100 expression tested by qRT-PCR was comparable to C57BL/6 wild-type (Figure 4A).

The offsprings with the genotype of miR-100^flox/flox^Alb-Cre^+^ mice were born alive and appeared healthy at birth (Figure 4B). We weighed pups every 3 days and found that no impact of liver-specific miR-100 deletion on the growth rate compared to their littermate controls (miR-100^flox/flox^) (Figure 4C). There was no obvious abnormality in the appearance of the liver from 10-week-old miR-100^flox/flox^ Alb-Cre^+^ mice (Figure 4D). Histological analysis by HE staining of the liver did not reveal significant pathological changes (Figure 4E). Consistently, we didn't observe the loss of miR-100 impaired liver function as indicated by serum AST and ALT levels (Figure 4F). The miR-100^flox/flox^ Alb-Cre^+^ livers weighed 1.63 ± 0.5 g on average, representing 5.74% of whole body weight. The liver weight per body weight (LW/BW) ratio was also comparable to their controls (Figure 4G).These initial data indicated that liver-specific knockout miR-100 has no significant effect on mouse phenotype, especially on the liver histology. MiR-100^flox/flox^Alb-Cre^+^ exhibited no substantial differences in the number of circulating red blood cells, white blood cells, lymphocytes, granulocytes, and platelets according to peripheral blood counts, relative to the abundance of these cells in their miR-100^flox/flox^ control littermates (Supplementary Figure 3).

**Figure 5:**
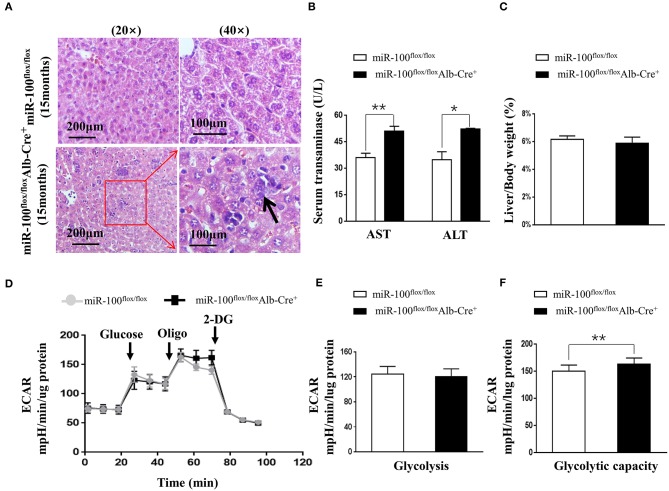
Phenotype analysis of the aged (15 months) hepatocyte-specific miR-100 deletion mice. **(A)** Representative HE staining of liver sections from each group were shown. Scale bars represent 200 μm (left) and 100 μm (right). Black arrows indicate inflammation foci and the hepatocytes with the larger nuclei. **(B)** Transaminase activity and **(C)** LW/BW ratios of miR-100^flox/flox^ Alb-Cre^+^ and miR-100^flox/flox^ littermates were measured. **(D–F)** ECAR were measured in real time in a Seahorse XF bioenergetic assay. **(D)** Average ECAR was calculated during the treatment of glucose, oligomycin, 2-DG as indicated. **(E)** Glycolysis and **(F)** glycolysis capacity were indicated. A total of 3 × 10^4^ hepatocytes from the 15-months-old miR-100 knock-out mice and control mice (*n* = 3) seeded for 24 h. Mean ± SEM is representative of 3 independent experiments carried out in triplicate. *P < 0.05, **P < 0.01 shows significant difference from the control mice after addition of oligomycin (Student's unpaired *t* test, two-tailed).

To investigate whether knockout of miR-100 lead to the malignant transformation of the hepatocytes in aged mice, we scarified the aged mice (15 months). Surprisingly, HE staining of the liver clearly revealed the infiltration of inflammatory cells and some hepatocytes with enlarged nuclei (Figure 5A) in miR-100 knockout mice, despite that no macroscopic HCC lesion was observed. Consistent with the inflammation, liver function was impaired in miR-100 knockout mice as indicated by increased serum AST and ALT levels (Figure 5B, 50.9 ± 3.5 vs. 35.9 ± 2.4, ^**^P < 0.01; 52.3 ± 1.5 vs. 34.8 ± 1.9, ^*^P < 0.05, respectively). There is no obvious impact of on the ratio of LW/BW (Figure 5C). Recent studies of metabolism support the concepts that metabolic adaptation is a primary aspect of transformation resulting from mutations in oncogenes and tumor suppressors. Therefore, we analyzed metabolic profiles of the hepatocytes from the mice (Figures 5D–F). As expected, the miR-100 knockout hepatocytes tend to adopt glycolysis. Extracellular acidification rate (ECAR) evaluation demonstrated that the miR-100 knockout hepatocytes generated more extracellular lactate (higher ECAR) compared with control cells (Figures 5D,F). Collectively, these results suggested that inactivation of miR-100 alone can induce the early malignant transformation of hepatocytes including local inflammation, morphological changes of hepatocytes, and glycolysis-biased metabolic phenotype.

### miR-100-Knockout Livers Displayed Up-Regulation of IGF1R-β and SHP-2

**Figure 6:**
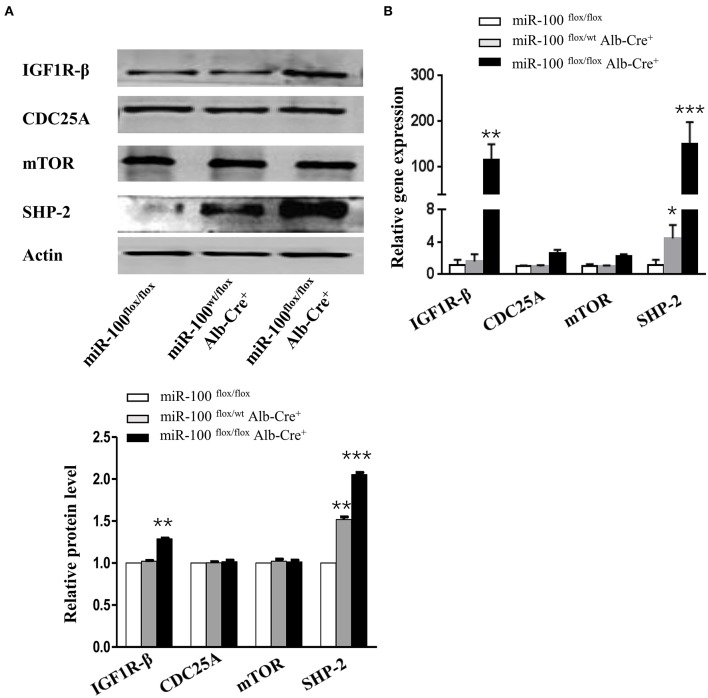
Increased expression of IGF1R-β and SHP-2 in miR-100-knockout livers. Western blot analysis **(A)** and quantitative RT-PCR analysis **(B)** of the expression of miR-100 target genes and SHP-2 in the livers from miR-100^flox/flox^ Alb-Cre^+^ and control littermates. Anti-β-actin was used as a loading control. Data are from three independent experiments with similar results. *P < 0.05, **P < 0.01, ***P < 0.001 (Student's unpaired *t* test, two-tailed).

mTOR (30, 31), IGF1R (31), and CDC25A (32) have been reported as the direct targets of miR-100 *in vitro* (Supplementary Figure 2), but whether these genes are targets of miR-100 *in vivo* is still unclear. IGF1R-β, mTOR and CDC25A were tested by qRT-PCR and Western blot. We observed a slight increase of IGF1R-β protein in the liver from miR-100^flox/flox^ Alb-Cre^+^ mice. However, mTOR and CDC-25A were not increased in the knockout livers (Figure 6A). Quantitative RT-PCR amplification of these genes displayed similar results, except that IGF1R-β was significantly elevated in homozygous liver (Figure 6B). SHP-2 was reported to be involved in HCC (24, 33). Furthermore, a significant up-regulation of SHP-2 protein was shown consistently in L02 and BEAS-2B cell lines (normal hepatocytes cell line and bronchial epithelial cell line, respectively) transfection with miR-100 inhibitor (unpublished data). We wondered whether miR-100 knockout would lead to the same change *in vivo*. SHP-2 expression was test by qRT-PCR and Western blot in the liver from miR-100^flox/flox^ Alb-Cre^+^ mice and the control mice. In agree with the findings from the *in vitro* experiments, SHP-2 expression increased both in mRNA and in protein level in miR-100 knockout mice (Figures 6A,B).

## Discussion

MiR-100 plays essential roles in carcinogenesis (9–12). Recent studies indicated that miR-100 is a context dependent miRNA, which can be over-expressed or under-expressed in different cancers and plays dual roles (13–22, 34). Increasing evidence shows that miR-100, a potential tumor suppressor, is downregulated in human HCC tissues (13, 14, 25, 35). miR-100 is found to be involved in multiple biological behavior of HCC cells, such as cell proliferation, apoptosis, migration, invasion (13) and autophagy (35). Liver specific knockout of miR-100 mice should be useful for gaining better understanding functions of this gene *in vivo* under different conditions.

Recent years, the development and application of Cre/Loxp system has already proven to be a powerful genetic modification tool. In particular, when Cre is expressed in mice harboring a LoxP-containing target gene, the desired gene modification can be restricted to certain cell types or developmental stages of the mouse depending on the cell specificity and timing of recombinase expression (36). The Alb promoter driving Cre is used for hepatocytes-specific gene deletion. We first report the generation of liver-specific deletion of miR-100 mouse line by crossing miR-100^flox/flox^ mouse with transgenic Alb-Cre mice. These knockout mice were confirmed by q-PCR assay of miR-100 expression in livers.

Liver miR-100-knockout pups are born alive and appear normally indicating no embryonic lethality. Natural death of the liver mir-100 knockout mice has not been observed up to 12 months of age.

Interestingly, we observed the glycolytic bias phenotype and larger nuclei in the hepatocytes from miR-100 knockout aged mice. These alterations as well as inflammation foci in the livers may be early malignant transformation of HCC. Recent evidence suggests that the energy metabolism alterations (especially a strong glycolytic metabolism) are primary features selected for during tumorigenesis (37) and have been included as a core hallmark of all cancer cells (38). Activation of the PI3K/Akt signaling pathway is probably the most common in spontaneous human cancers. Activated PI3K/Akt was found to result in enhanced glycolysis (39). Thus, we have reason to believe that over time, miR-100 knockout mice will induce spontaneous liver cancer.

Although there are plethoras of target genes of miR-100 discovered by biological information analysis and *in vitro* systems, it is largely unknown which of them have a causative role in the hepatocarcinoma genic process *in vivo*. This mouse model is optimal for determining changes in the expression profile of miR-100 target genes in the context of miR-100 deletion *in vivo*. Our data indicated that hepatic miR-100 knockout resulted in a significant increase in IGF1R-β. Other target genes such as CDC25A, mTOR were not affected, although miR-100 was reported to cooperate with other factors to down-regulate mTOR pathway in prostate cancer (40). Then *in vivo* data support that IGF1R-β may be more potential target in the liver tissue, by which miR-100 act as a tumor suppressor in HCC. Over-expression of the IGF-1R is the most common findings associated with deregulated IGF signaling in human cancers (41).

Of note, we observed a statistically significant increase in SHP-2 expression in the miR-100 knockout liver. SHP-2 is the first reported proto-oncogene encoding protein tyrosine phosphatase (PTP) (42). Gain-of-function mutations of this gene were found in leukemias (42, 43). Han et al. reported that SHP-2 was up-regulated in 65.9% of human HCCs and contributed to HCC progression. Inhibition of SHP-2 expression suppressed the growth of HCC xenografts in mice (24). Increased expression of SHP2 was also reported to be detected in recurrent and chemoresistant HCCs patients. Moreover, increased SHP2 facilitated liver cancer stem cell (CSC) expansion by augmenting the dedifferentiation of hepatoma cells and enhancing the self-renewal of liver CSCs. Mechanistically, high expression of SHP-2 over-activated β-catenin pathway thereby resulted in stem cell expansion (33). Although it was reported that SHP-2 negatively regulate IGF1R phosphorylation (44, 45), the underlying mechanism, however, responsible for increased SHP-2 expression by miR-100 deletion need to be further elucidated. It's possible that determining the regulatory relationship between SHP-2 and IGF1R can better clarify the mechanism of miR-100 inactivation promoting the occurrence and development of HCC.

The HCC genesis is a long-term process, which arises from the substantial accumulation of genetic and/or epigenetic alterations. The process is also influenced by environmental risk factors. Our data indicated that the inactivation of miR-100 alone in mice fails to result in the spontaneous visible HCC (<15 months). This may be explained by the fact that the importance of gene- environment interplay on tumorigenesis. Alternatively, the time (<15months) is not enough for spontaneous HCC genesis.

In summary, we have generated a liver-specific knockout of the miR-100 gene mouse model which will be valuable for further studies of HCC. Also, this animal model could be widely used for exploring miR-100 biologic function in the liver, especially in hepatocytes, and miR-100 related liver diseases as well as novel medication development.

## Ethics Statement

This study was carried out in accordance with the Anhui Medical University Institutional Animal Care and Use Committee (IACUC), The protocol was approved by the Anhui Medical University Institutional Animal Care and Use Committee (IACUC).

## Author Contributions

YZ and SW designed research. DY, ST, YY, FY, and WJ performed research. DY, YY, HF, YL, and FZ analyzed the data. DY and YZ wrote the paper. HF revised the manuscript. ST provided the HE staining, AST&ALT level testing, and liver/body weight monitoring in aged miRNA100 knock-out mice during the revision (Figures 5A-C). FY performed hepatocytes isolation and seahorse metabolic flux analysis of hepatocytes in miRNA-100 knock-out mice (Figures 5D-F).

### Conflict of Interest Statement

The authors declare that the research was conducted in the absence of any commercial or financial relationships that could be construed as a potential conflict of interest.
