# Supplementary material for: Generation and Validation of miR-100 Hepatocyte-Specific Knock-Out Mice
Source: Front Oncol. 2019 Jun 26;9:535. doi: 10.3389/fonc.2019.00535 (PMC6606737; doi:10.3389/fonc.2019.00535)
Supplement: Supplementary file 1 [file Data_Sheet_1.PDF]

Supplementary Figure 1

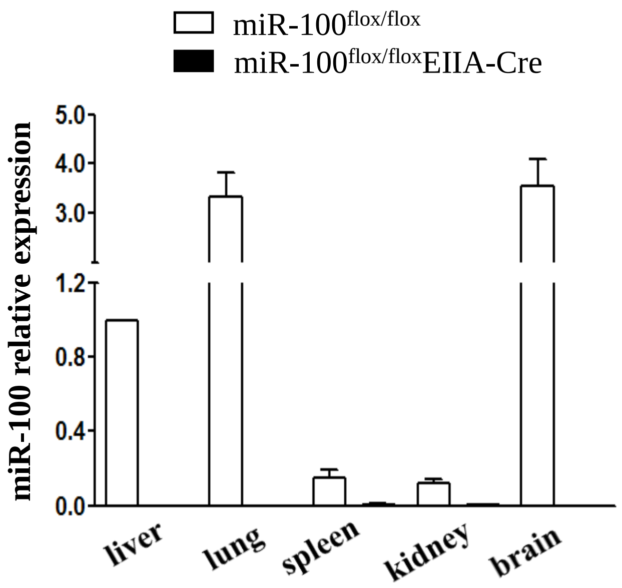

Supplementary Figure 1. MiR-100 expression in organs of miR-$100^{flox/flox}$ and miR-$100^{flox/flox}$ EIIa-Cre mice. QRT-PCR analysis of the expression of miR-100 in liver, lung, spleen, kidney and brain of mice. The expression level was normalized and are presented relative to the level expressed in the liver.
