# Supplementary material for: Generation and Validation of miR-100 Hepatocyte-Specific Knock-Out Mice
Source: Front Oncol. 2019 Jun 26;9:535. doi: 10.3389/fonc.2019.00535 (PMC6606737; doi:10.3389/fonc.2019.00535)
Supplement: Supplementary file 2 [file Data_Sheet_2.PDF]

Supplementary Figure 2

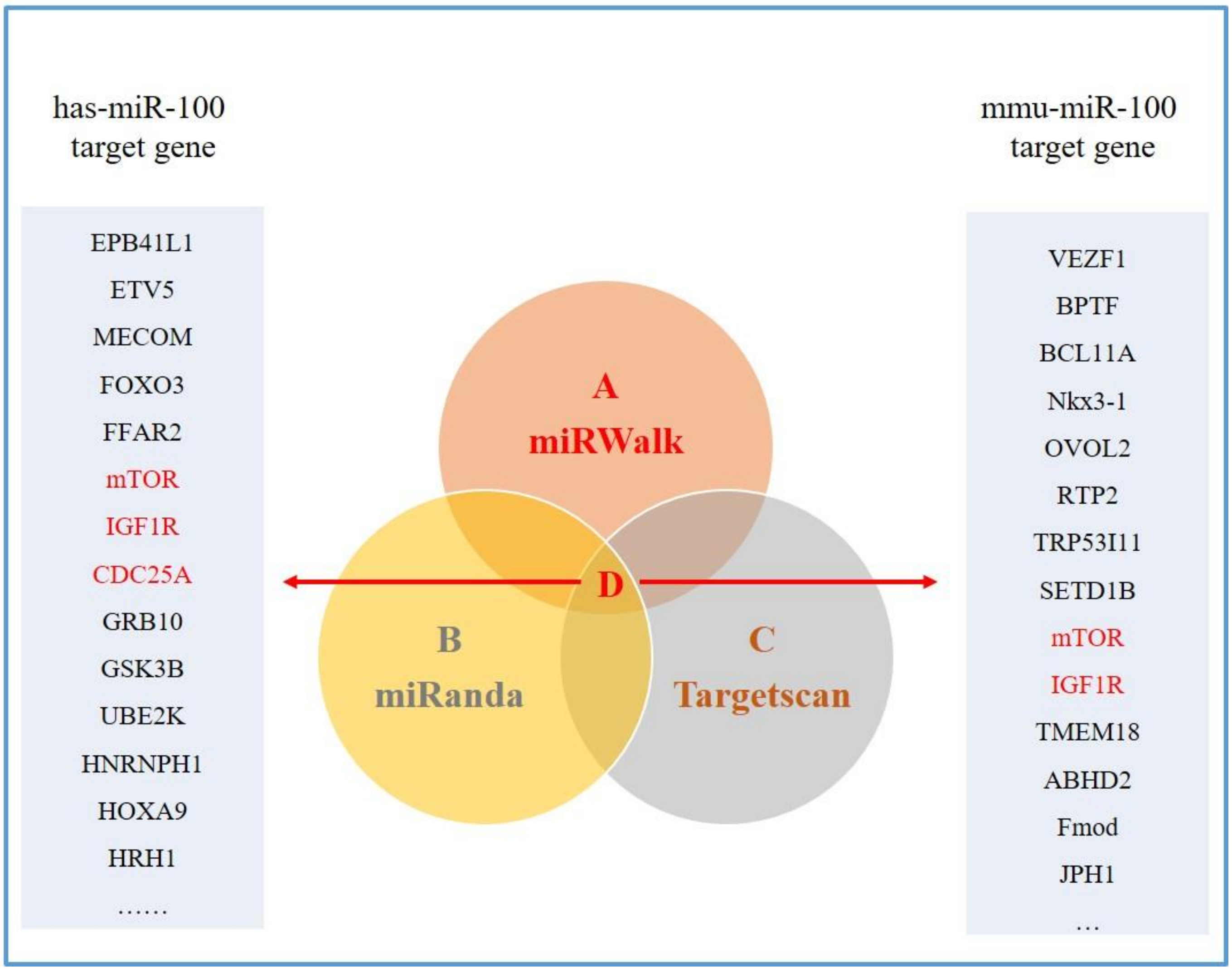

Supplementary Figure 2. Predicted targets of mouse and human miR-100 using miRWalk, miRanda and Target Scan software. IGF1R and mTOR are targets of mouse/human miR-100-5p. CDC25A is a target of human miR-100-3p.
