# Supplementary material for: Generation and Validation of miR-100 Hepatocyte-Specific Knock-Out Mice
Source: Front Oncol. 2019 Jun 26;9:535. doi: 10.3389/fonc.2019.00535 (PMC6606737; doi:10.3389/fonc.2019.00535)
Supplement: Supplementary file 3 [file Data_Sheet_3.PDF]

Supplementary Figure 3

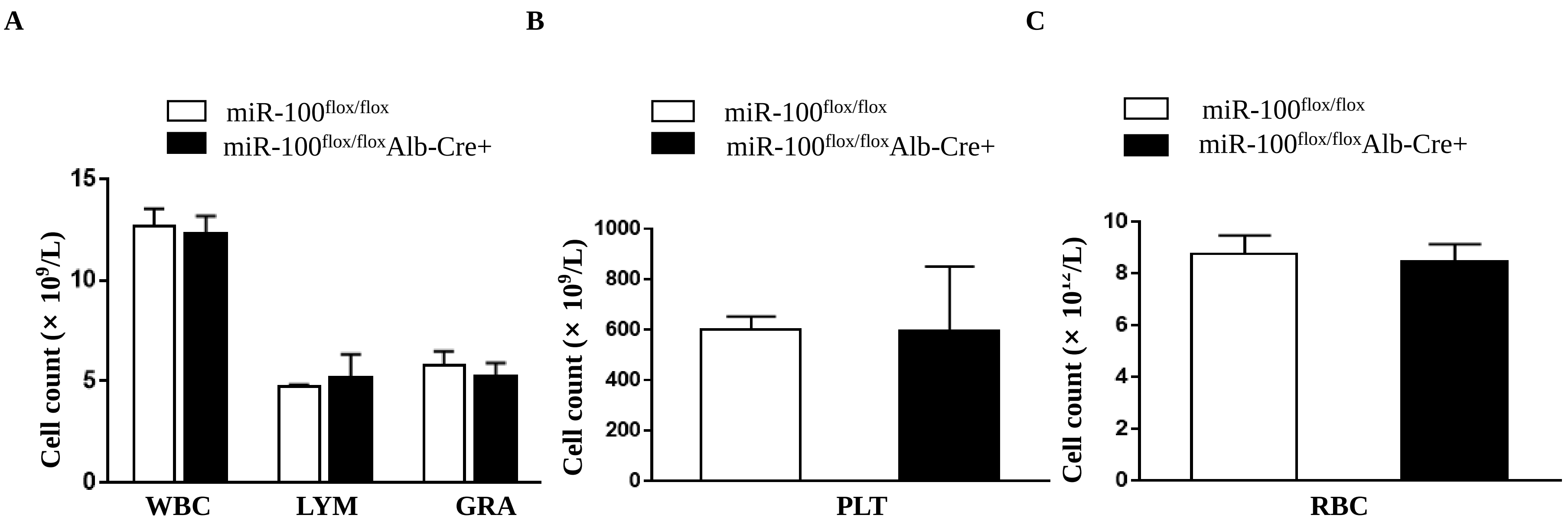

Supplementary Figure 3. White blood cell (WBC), circulating lymphocyte (LYM), granulocyte (GRA), **(A)** platelet counts (PLT) **(B)** and red blood cells (RBC) **(C)** of miR-100$^{flox/flox}$ and miR-100$^{flox/flox}$ Alb-Cre^{+} mice. n=3.
